# Automated EHR score to predict COVID-19 outcomes at US Department of Veterans Affairs

**DOI:** 10.1371/journal.pone.0236554

**Published:** 2020-07-27

**Authors:** Thomas F. Osborne, Zachary P. Veigulis, David M. Arreola, Eliane Röösli, Catherine M. Curtin

**Affiliations:** 1 US Department of Veterans Affairs, Palo Alto Healthcare System, Palo Alto, California, United States of America; 2 Department of Radiology, Stanford University School of Medicine, Stanford, California, United States of America; 3 US Department of Veterans Affairs, Central Iowa Health Care System, Des Moines, Iowa, United States of America; 4 Department of Medicine, Stanford University School of Medicine, Stanford, California, United States of America; 5 Department of Surgery, Stanford University School of Medicine, Stanford, California, United States of America; BronxCare Health System, Affiliated with Icahn School of Medicine at Mount Sinai, NY, UNITED STATES

## Abstract

The sudden emergence of COVID-19 has brought significant challenges to the care of Veterans. An improved ability to predict a patient’s clinical course would facilitate optimal care decisions, resource allocation, family counseling, and strategies for safely easing distancing restrictions. The Care Assessment Need (CAN) score is an existing risk assessment tool within the Veterans Health Administration (VA), and produces a score from 0 to 99, with a higher score correlating to a greater risk. The model was originally designed for the nonacute outpatient setting and is automatically calculated from structured data variables in the electronic health record. This multisite retrospective study of 6591 Veterans diagnosed with COVID-19 from March 2, 2020 to May 26, 2020 was designed to assess the utility of repurposing the CAN score as objective and automated risk assessment tool to promptly enhance clinical decision making for Veterans diagnosed with COVID-19. We performed bivariate analyses on the dichotomized CAN 1-year mortality score (high vs. low risk) and each patient outcome using Chi-square tests of independence. Logistic regression models using the continuous CAN score were fit to assess its predictive power for outcomes of interest. Results demonstrated that a CAN score greater than 50 was significantly associated with the following outcomes after positive COVID-19 test: hospital admission (OR 4.6), prolonged hospital stay (OR 4.5), ICU admission (3.1), prolonged ICU stay (OR 2.9), mechanical ventilation (OR 2.6), and mortality (OR 7.2). Repurposing the CAN score offers an efficient way to risk-stratify COVID-19 Veterans. As a result of the compelling statistical results, and automation, this tool is well positioned for broad use across the VA to enhance clinical decision-making.

## Introduction

COVID-19 has disrupted our healthcare system through its rapid emergence, extreme variation in presentation, and potentially devastating consequences. Improved understanding of the likely clinical course for each individual patient would allow frontline staff and executive leadership to proactively align resources, optimize location of care decisions, and assist with strategies to safely ease physical distancing.

The Veterans Health Administration (VA) developed a risk stratification tool, the Care Assessment Need (CAN) score, to support ambulatory care teams in the systematic identification of high-risk outpatients to better target resources. The latest CAN scores use binary logistic regression probability models to produce a single cumulative score from 0 to 99, with higher CAN score representing a greater risk patient compared to their peers. CAN scores are automatically calculated from the VA’s national electronic health record (EHR) database, utilizing multiple structured data points, including socio-demographics, clinical diagnoses, vital signs, medications, lab values, and health care utilization data. The scores are updated weekly on all living Veterans who receive primary care services within VA [1,2]. The CAN score models are calculated for six possible event categories; hospitalization, mortality, and mortality or hospitalization, for 1-year and 90-day time periods. In the latest iteration of the CAN (version 2.5), inpatient Veterans also have CAN mortality models generated [3]. Our recent work has also demonstrated that the CAN score can be repurposed as a presurgical risk assessment tool [4].

This quality improvement project was designed to assess if the CAN score could be utilized to rapidly improve the ability to risk-stratify Veterans diagnosed with COVID-19 and enhance existing care strategies.

## Materials and methods

This quality improvement study received a Determination of Non-Research from Stanford IRB (Stanford University, Stanford, CA, USA).

VA is the largest integrated health care system in the United States and offers care at 1,255 health care facilities for over 9 million enrolled Veterans [5]. This study utilized the VA Corporate Data Warehouse (CDW), a central repository that aggregates VA EHR data nightly. Patients diagnosed with COVID-19 from March 2, 2020 to May 26, 2020 were identified by positive COVID-19 Polymerase Chain Reaction (PCR) lab results administered by the VA.

For this study, we utilized the preadmission CAN 1-year mortality model version 2.5, which was recorded closest in time prior to the Veterans first positive COVID-19 lab test, but no later than 6 months prior to the lab administration. The mortality model was chosen as it is the only model calculated on inpatient Veterans, and the 1-year CAN score was chosen over its 90-day equivalent as it showed consistent superior performance in a sensitivity analysis for this study.

Six key patient outcomes of interest were evaluated: hospital admission, prolonged hospital stay (greater than 5 days), ICU admission, prolonged ICU stay (greater than 3 days), mechanical ventilation defined by ventilator codes (S1 Table), and mortality.

**Fig 1:**
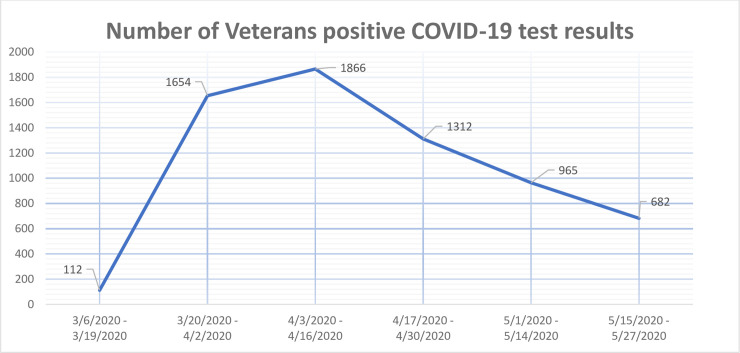
Number of Veterans in the cohort with positive COVID-19 test results per week.

The number of Veterans with a positive COVID-19 test result during the study period shows an early peak and a subsequent steady decline (Fig 1). Because the outcomes of the more recently diagnosed patients in our cohort have not yet been observed due to the progressive nature of the disease, there is a general underestimation of the outcome risks. Therefore, when more information is available, we would expect an even more pronounced statistical association with the CAN score. However, due to the recent emergence of the disease and still undefined observation window for COVID-19 outcomes, we did not exclude patients based on time of diagnosis.

### Analysis

We performed a median split on the cohort’s CAN score to construct a binary variable of high CAN score yes/no. We then performed bivariate analyses with the explanatory variable and each outcome variable using Chi-square tests of independence to assess the presence and strength of association between those two quantities.

Additionally, logistic regression models using the continuous CAN score as the sole covariate were fitted to predict each binary outcome individually. The models have been trained on 70% of the available data and validated on the remaining 30% to assess the discriminatory power captured by the concordance (or c) statistic. The models were then retrained on the full dataset to report final model parameters, confidence intervals, p-values and pseudo-R2.

Demographic predictors (e.g. age, gender) are already incorporated in the CAN score calculations and thus were not included in our models. Analyses were performed using Python 3.7.

## Results

As of May 26, 2020, we identified 9265 Veterans with COVID-19 by PCR lab test results performed at VA. Of this group, 6591 patients had a CAN score within six months of a positive COVID-19 lab test and made up our final cohort. The overall patient age and gender in the cohort were consistent with the VA population (Table 1). The disproportionally large number of African American men diagnosed with COVID-19 in our cohort is also similar to another recently reported VA study [6].

**Table 1:** Characteristics of Veterans identified with a positive COVID -19 diagnosis by lab test at VA, and CAN score, from March 2, 2020 to May 26, 2020.

	All Patients in Cohort	Patients with CAN ≤ 50	Patients with CAN > 50
Characteristic	n = 6591	n = 3376	n = 3215
**CAN—Mortality 1 Year (Mean) (SD)**	52.1 (32.3)	24.2 (15.7)	81.4 (14.5)
**Male (%)**	5996 (91%)	2848 (84.4%)	3148 (97.9%)
**Age (Mean) (SD)**	61.9 (15.5)	51.7 (12.7)	72.6 (10)
**Hypertension**	4679 (71%)	1886 (55.9%)	2793 (86.9%)
**COPD**	2733 (41.5%)	962 (28.5%)	1771 (55.1%)
**CHF**	1405 (21.3%)	200 (5.9%)	1205 (37.5%)
**Diabetes**	2910 (44.2%)	1011 (30%)	1899 (59.1%)
**Charlson Comorbidity Index (CCI) (SD)**	3.7 (3.7)	1.66 (2.2)	5.82 (3.8)
**AMERICAN INDIAN, ALASKA NATIVE OR OTHER PACIFIC ISLANDER**	116 (1.8%)	60 (1.8%)	56 (1.7%)
**ASIAN**	74 (1.1%)	58 (1.7%)	16 (0.5%)
**BLACK OR AFRICAN AMERICAN**	3162 (47.8%)	1689 (50%)	1473 (45.8%)
**WHITE**	2823 (42.8%)	1312 (38.9%)	1511 (47%)
**DECLINED TO ANSWER**	268 (4.1%)	173 (5.1%)	95 (3%)
**UNKNOWN BY PATIENT**	76 (1.2%)	41 (1.2%)	35 (1.1%)
**NOT REPORTED**	72 (1.1%)	43 (1.3%)	29 (0.9%)

Within our cohort, 2868 COVID-19 positive Veterans required hospital care (44%). Of those who required hospital care, 1868 had a prolonged hospital stay (28%). For those 2868 Veterans admitted to the hospital, 782 required ICU care (27%), and of those ICU patients, 574 required prolonged ICU stay (73%). Of the total cohort, 318 Veterans required mechanical ventilation (5%) and 634 died (10%). During this short evaluation period, 330 Veterans were readmitted (5%).

The median CAN score of the cohort was 50, and we utilized a median split designating a high CAN score as greater than 50. A high CAN score imparted a large significant increase in odds of all adverse outcomes for Veterans diagnosed with COVID-19 (Table 2). Results demonstrate that a COVID-19 positive Veteran, randomly selected from the high CAN score group, is on average 7 times more likely to die from COVID-19 than a Veteran with a CAN score below 50. These results are further supported by the logistic regression models demonstrating that the CAN score captures meaningful information of statistical significance to predict all studied patient outcomes (Table 3). As expected, a higher CAN score is associated with an increased likelihood in any outcome we assessed, with mortality being the most strongly affected: The odds of a lethal outcome increased by roughly 3.8% for every unit-increase in the CAN score. Based on the c-statistics, mechanical ventilation (0.63) and outcomes associated with an ICU stay (both 0.68 and 0.69) are the most difficult to solely predict from the CAN score results for our cohort, whereas mortality prognoses (0.8) is the most reliable.

**Table 2:** Odds ratios for adverse outcomes in Veterans diagnosed with COVID-19 and a CAN score greater than 50.

Dependent Variable	Odds Ratio	95% CI
**Hospital Admission**	4.6	4.11–5.07
**Long Stay (> 5 Days)**	4.5	3.98–5.05
**ICU Admission**	3.1	2.61–3.63
**ICU Long Stay (>3 Days)**	2.9	2.37–3.46
**Mechanical Ventilation**	2.6	2.01–3.29
**Death**	7.2	5.77–9.08

Odds Ratio p values for all outcomes are < .001

**Table 3:** Results of logistic regression model parameters for CAN score greater than 50.

Dependent Variable	Constant	Coef	OR	CI 95% Lower	CI 95% Upper	C-Stat.	R2
**Hospital Admission**	-1.7	0.028	1.028	0.026	0.029	0.73	0.12
**Long Stay (> 5 Days)**	-2.5	0.028	1.028	0.026	0.030	0.72	0.11
**ICU Admission**	-3.15	0.019	1.019	0.017	0.022	0.69	0.05
**ICU Long Stay (>3 Days)**	-3.4	0.018	1.018	0.015	0.021	0.68	0.04
**Mechanical Ventilation**	-4	0.018	1.018	0.014	0.021	0.63	0.03
**Death**	-4.8	0.038	1.039	0.034	0.042	0.8	0.14

p values for all outcomes are < .001

## Discussion

There is extreme variation in clinical presentation and course of those diagnosed with COVID-19 [7,8]. Many of the factors that are thought to contribute to this variation, (comorbidities, demographics, medications etc.) are available as structured elements in the EHR [9,10,11]. These same variables are also found to be associated with decreased survival rate and need for intensive care for COVID-19 patients [12]. Being able to quickly and efficiently consolidate the many preexisting patient factors into a single risk score would assist time-constrained clinicians in their assessment and management decisions. Fortunately, these EHR variables are already consolidated in an automated CAN score at VA.

This study demonstrated that a high CAN score is associated with increased odds of adverse clinical outcomes for Veterans with COVID-19. There are several ways the CAN score could be repurposed as a clinical decision support tool to help inform the optimal management of Veterans with COVID-19. For example, a COVID-19 positive Veteran with a high CAN score could be flagged for closer monitoring and more aggressive initial care. This pandemic has emphasized that early advanced care discussions with patients and families is of high importance, and unfortunately, these discussions often do not occur [13,14]. However, a high CAN score could provide a flag to proactively initiate early advanced care discussions.

Currently there are broad discussions about how to safely ease societal physical distancing measures [15]. The CAN score could be utilized as a complementary metric to help identify Veterans at highest risk for poor outcomes if infected with COVID-19, leading to a more personalized approach to physical distancing restrictions and supportive resources. For example, a high CAN score Veteran may be more appropriately triaged to telehealth before considering a routine in-person clinic visit. The CAN score could also identify large sub-populations of Veterans in greatest need of long-term remote technology tools, resources, and support. Therefore, an efficiently calculated risk tool, such as the CAN score, is positioned to promptly identify how to strategically redirect VA resources and aide in the development and deployment of remote care tools to optimize the health of specific populations of Veterans. In addition, our study added to the emerging literature showing that African American men are disproportionately diagnosed with COVID-19. This requires more work to better understand the drivers of this important disparity.

There are some cautions with this study. First, as with any risk score, healthcare teams should always take a single numerical value in clinical context and consider all other available information in decision making. In addition, our study utilized a CAN score of 50 for the initial evaluation and we may find that an optimal CAN score threshold, for each clinical endpoint, may be different as a result of newly acquired data and insight from clinical staff.

An important limitation of our work is that the recent emergence of the disease means that there is an incomplete timeline of information and therefore insights will be refined as the pandemic evolves. However, this evaluation included a large cohort of patients, from in a wide geographic range, utilizing a robust longitudinal database. Over the next few months, the data quality and availability of the COVID-19 patient outcomes will improve steadily, offering the opportunity to further refine our data analysis framework and gather even stronger support for the use of the CAN score for risk-stratification purposes in the battle against COVID-19. An additional strength is that CAN score is a readily available automated risk score, and therefore extremely efficient, for time-limited VA clinicians. Since the CAN model draws from an already available set of variables in the EHR, it produces an immediately accessible and objective score, with the opportunity for more consistent patient risk assessment.

## Conclusion

The VA’s CAN score offers an efficient way to utilize existing EHR data to predict the clinical course, and promptly facilitate management decisions, for COVID-19 Veterans.

## Supporting information

S1 Table VA ventilator codes utilized to identify intubated Veterans. (DOCX)
