# Supplementary material for: Automated EHR score to predict COVID-19 outcomes at US Department of Veterans Affairs
Source: PLoS One. 2020 Jul 27;15(7):e0236554. doi: 10.1371/journal.pone.0236554 (PMC7384633; doi:10.1371/journal.pone.0236554)
Supplement: S1 Table — (DOCX) [file pone.0236554.s001.docx]

| **S1 Table. VA ventilator codes utilized to identify intubated Veterans**  **Orderable Item Name** |
| --- |
| 5W-VENTILATOR ORDERS |
| Adjust Ventilator |
| AL VENTILATOR |
| BK RESPIRATORY VENTILATOR INPT |
| BMS VENTILATOR |
| BMS VENTILATOR SETTINGS - RTX |
| HAND-HELD NEBULIZER/MDI FOR VENTILATORS |
| Initial Ventilator Settings |
| INVASIVE VENTILATOR INITIATION & MGMT |
| Mech. Ventilator |
| MEDICINE (RT VENTILATOR NEW) |
| NON-INVASIVE MASK VENTILATOR |
| NON-INVASIVE VENTILATOR INITIATION & MGMT |
| NY RESPIRATORY VENTILATOR INPT |
| NY VENTILATOR (NEW) INPT |
| ORZ-MU RT OI VENTILATOR CHANGE PEEP |
| ORZ-MU RT OI VENTILATOR ORDER |
| PRVC Ventilator Settings |
| RESP THERAPY MECHANICAL VENTILATOR INPT |
| RESP VENTILATOR |
| RESPIRATORY CONSULT/ORDERS INPATIENT - VENTILATOR MANAGEMENT |
| RT Ventilator Change ALM |
| RT VOLUME VENTILATOR MICU/CCU |
| RT VOLUME VENTILATOR SICU/TICU |
| VENTILATOR |
| VENTILATOR (INVASIVE) |
| Ventilator - CMV Mode |
| VENTILATOR - VOLUME CONTROLLED |
| Ventilator Assoc. Pneumonia Prevention protocol |
| Ventilator Change Order [Omaha] |
| VENTILATOR MODE: VC/AC |
| Ventilator Order |
| VENTILATOR ORDERS |
| VENTILATOR ORDERS INPT |
| VENTILATOR ORDERS: Initiate ARDSNet Protocol |
| VENTILATOR PARAMETER |
| Ventilator Sedation/Analgesia Protocol |
| Ventilator Settings |
| VENTILATOR STL |
| While on Ventilator, maintain sedation for RASS goal 0 to 1 |
| ZZENDOTRACHEAL TUBE |
| ZZVentilator Change Order [Omaha] |
